# Potential of Bioremediation and PGP Traits in *Streptomyces* as Strategies for Bio-Reclamation of Salt-Affected Soils for Agriculture

**DOI:** 10.3390/pathogens9020117

**Published:** 2020-02-13

**Authors:** Neli Romano-Armada, María Florencia Yañez-Yazlle, Verónica P. Irazusta, Verónica B. Rajal, Norma B. Moraga

**Affiliations:** 1 Instituto de Investigaciones para la Industria Química (INIQUI), Universidad Nacional de Salta (UNSa)-Consejo Nacional de Investigaciones Científicas y Técnicas (CONICET). Av. Bolivia 5150, Salta 4400, Argentina; nelir000@gmail.com (N.R.-A.); florenciayanez93@gmail.com (M.F.Y.-Y.); irazustaveronica@gmail.com (V.P.I.); normoraga@gmail.com (N.B.M.); 2 Facultad de Ingeniería, UNSa, Salta 4400, Argentina; 3 Facultad de Ciencias Naturales, UNSa, Salta 4400, Argentina; 4 Singapore Centre for Environmental Life Sciences Engineering (SCELSE), School of Biological Sciences, Nanyang Technological University, Singapore 639798, Singapore

**Keywords:** actinomycetes, bioremediation, boron compounds, salt-affected soils, plant growth-promoting (PGP)

## Abstract

Environmental limitations influence food production and distribution, adding up to global problems like world hunger. Conditions caused by climate change require global efforts to be improved, but others like soil degradation demand local management. For many years, saline soils were not a problem; indeed, natural salinity shaped different biomes around the world. However, overall saline soils present adverse conditions for plant growth, which then translate into limitations for agriculture. Shortage on the surface of productive land, either due to depletion of arable land or to soil degradation, represents a threat to the growing worldwide population. Hence, the need to use degraded land leads scientists to think of recovery alternatives. In the case of salt-affected soils (naturally occurring or human-made), which are traditionally washed or amended with calcium salts, bio-reclamation via microbiome presents itself as an innovative and environmentally friendly option. Due to their low pathogenicity, endurance to adverse environmental conditions, and production of a wide variety of secondary metabolic compounds, members of the genus *Streptomyces* are good candidates for bio-reclamation of salt-affected soils. Thus, plant growth promotion and soil bioremediation strategies combine to overcome biotic and abiotic stressors, providing green management options for agriculture in the near future.

## 1. Introduction

Bacteria and Archaea are domains of unicellular prokaryotic microorganisms, which inhabit diverse soil ecosystems, from warm and humid places, densely populated with vegetation, to deserts and barren sites, even in the highest latitudes. Despite their small size, and due to their versatile metabolic capacities, they play a crucial role in the matter cycle by decomposing organic matter, degrading compounds, transforming mineral compounds, treating wastes, and participating in a variety of symbiotic reactions with plants, animals, and other soil organisms [1].

The composition of microbial populations in soils varies with many factors such as type of soil, pH, moisture content, aeration, relative composition of soil constituents, climatic conditions, and relationships established between microorganisms, among others. However, the overall distribution of microorganisms varies [2], showing a two-fold increase in the number of colony forming units (CFU) per gram of dry soil for actinobacteria (10^6^ to 10^7^) in comparison with fungi (10^4^ to 10^5^), making the first the most abundant microorganisms in the soil [3]. 

Sporulated bacteria, actinobacteria, and fungi are resistant to drought. Sporulation is a mechanism induced by the lack of nutrients or by unfavorable environmental conditions that allows microorganisms to survive for long periods in dry environments and with a lack of nutrients. The aerial spores of most actinobacteria (Gram-positive bacteria) genera resist desiccation and show a slightly higher resistance to dry heat compared to that shown by Gram-negative bacteria [4]. High osmotic pressure, due to the low water activity (a_w_) of spores, seems to be particularly significant in the passive thermal resistance of bacterial spores, and also in osmotic stresses, where regulatory proteins respond to small molecule signals that serve to activate or repress the transcription of genes that allow the organism to survive [5]. Under saline stress, such as that of salt-affected soils, bacterial cells obtain osmotic signals and then absorb or synthesize many compatible solutes (e.g., glycine betaine, trehalose, and ectoin) to maintain an internal osmotic pressure equivalent to the external [5], thus avoiding irreversible damage. These processes regulate the level of transcription of osmoprotective transport and imply an instantaneous response to osmotic stress [6]. Moreover, K^+^ can regulate the transcription of a gene whose product is a DNA-binding protein that represses its transcription against osmotic stress (SCO3128-3130) [5].

### 1.1. The Genus Streptomyces

Conventional isolation methods show that the genus *Streptomyces* includes more than 95% of the filamentous actinobacteria in the soil [7,8]. They are mostly non-pathogenic [9], aerobic heterotrophic, spore-forming, and present high G + C content (70–74%) in their DNA. Although *Streptomyces* are slow-growing bacteria, they cover an essential niche by decomposing a wide variety of polymers produced by plants, fungi, and higher animals [10]. Due to their lytic enzymes, they can also degrade recalcitrant substances such as cellulose, lignocellulose, xylan, and lignin and organic material. Moreover, they have plant growth-promoting (PGP) properties, such as production of siderophores [11] and indole-3-acetic acid [12], in some cases enhancing the production of tomatoes, wheat [13], chickpea [14], sorghum, and rice [15,16]. Additionally, they are involved in the induction of resistance in different plant-pathogen systems [17]. *Streptomyces* have developed different strategies of persistence and maintenance of population in soils, such as cycles of rapid proliferation, sporulation, secretion of enzymes, and antibiotics production, among the most common [18]. 

The genus *Streptomyces* has been widely studied as a source of new antimicrobial agents, due to its production of a large number of antibiotics [19] and to the need to find new compounds to face the rising of drug-multiresistant pathogens [20]. Additionally, the capacity of *Streptomyces* to produce useful secondary metabolites [21], inspired increasing research to find new products and activities with biotechnological potential.

Regarding extreme environments, *Streptomyces* are involved in calcite biomineralization processes in marine sediments [22], and in the biomineralization of struvite [23] and formation of boron bio-precipitates, as a strategy to resist the toxic effect of boric acid (by immobilizing boron in these minerals) [6].

All these abilities make *Streptomyces* exceptional soil bioremediation agents, not only for their direct interaction with the environment but for promoting growth of plants that can ameliorate contaminated soils [15], for example, in salt contaminated soils. 

### 1.2. Streptomyces Life Cycle

**Figure 1:**
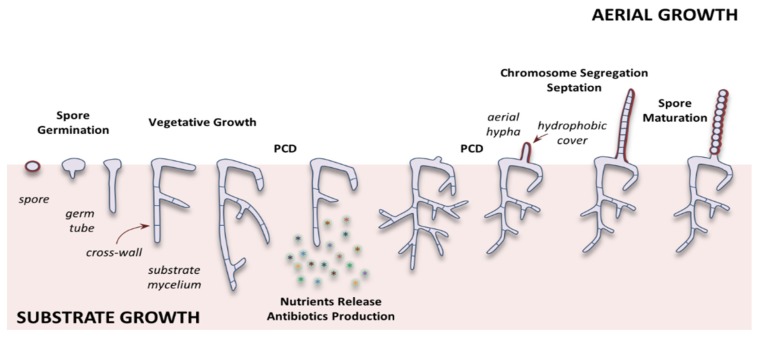
*Streptomyces* sporulation cycle on solid medium, when environmental conditions are optimal for spore germination. The germ tube elongates, and the vegetative cells show apical growth, separating in compartments connected by cross-walls. If environmental conditions are such that sporulation is induced, this first mass of substrate mycelia undergoes two rounds of programmed cell death (PCD)-like mechanisms. After the first PCD-like mechanism, the second mass of multinucleated mycelia without hydrophobic covers that allows nutrient transfer is produced. Following the second PCD-like mechanism, aerial hyphae with hydrophobic covers are produced, accompanied by the production of antibiotics to control the microorganisms attracted by PCD-like mechanism´s nutrients released into the growth medium. The aerial mycelium grows forming fluff-like colonies, and after hyphae septation and spore maturation, spores are released to start the cycle over again.

Starting the life cycle of *Streptomyces* with spore germination, apical growth follows after [24] (Figure 1), and hyphal branching results in a network of vegetative mycelium [25].

*Streptomyces* are a rare example of multicellular bacteria, where each compartment contains multiple copies of the chromosome [26,27]. During vegetative growth, cell division does not lead to cell fission; instead, by forming cross-walls the hyphae separates into connected compartments [28]. Cell division during vegetative growth results in the formation of widely spaced hyphal cross-walls, which delimit adjacent elongated compartments each one containing multiple copies of the genome. Vegetative mycelium hyphae differentiate from aerial’s ones when nutrient depletion occurs, a fact correlated with the temporal production of antibiotics [29]. The spongy white appearance of the colonies is due to aerial hyphae, which end up forming unigenomic spore chains, and differed from vegetative ones because they are twice as wide and do not have branches, besides their fast elongation rate and intensive chromosome replication [29]. The length between these vegetative cross-walls varies significantly, not only between different *Streptomyces* species but also in different growth conditions and mycelial ages [30].

Environmental stress conditions such as temperature, pH, availability of oxygen, and nutrients [31], presence of xenobiotics like heavy metals [32], and high salt concentrations, lead vegetative mycelia to differentiate and form aerial hyphae, which are erected sporogenic structures (Figure 1). In the face of stress, a programmed cell death (PCD)-like mechanism, leads vegetative mycelia to degrade by autolysis, to acquire the amino acids, amino sugars, nucleotides, and lipids required to build a second mass of aerial mycelium [33,34]. At this stage, the attraction of other motile competing microbes present in the habitat is inevitable [30]. Therefore, most antibiotics are produced at this moment as a defense and survival mechanism [35,36].

Two rounds of PCD-like mechanism occur during the life cycle of *Streptomyces*: after spore germination (a first compartmentalized mycelium grows out and then undergoes PCD-like mechanism), and during the onset of development (a second pre-sporulating multinucleated mycelium with no hydrophobic layer undergoes a PCD-like mechanism) [37] (Figure 1). At these stages, the hyphae (vegetative or substrate) are lysed to provide the nutrients required for the next growth of aerial mycelium. These aerial hyphae, which give the colonies their characteristic fluffy appearance, eventually differentiate to form chains of unigenomic spores [38].

Aerial hyphae have cells that do not branch as much as other hyphae. Besides, they are twice as wide and grow faster than vegetative hyphae. As other hyphae, those aerial present polar growths, i.e., by extension of the tip. Once sufficient aerial biomass is produced, a signal is transmitted (probably related to regulatory proteins of Whi, WhiA, and WhiB), resulting in cessation of growth, followed by the onset of sporulation [39]. It is known without much certainty that Whi regulatory proteins (WhiA and WhiB) are related to growth disruption. In addition, mutations in the respective *whiA* and *whiB* genes produce identical phenotypes of long aerial hyphae that do not initiate cell division [30]. The outer part of the aerial hyphae is surrounded by a hydrophobic sheath that later becomes part of the spore protective layer [40,41]. This configuration allows them to cross the surface layer with moist air from the soil [42], through a mechanism analogous to that proposed for fungi [43]. Another essential function of the sheath is the creation of a channel along the outer hyphal wall that can facilitate nutrients transport. This argument could explain why nutrients and other metabolites diffuse, from the vegetative hyphae in the basal part of the colony to the growing tips of the aerial hyphae, efficiently through long distances across the transverse walls [44].

**Figure 2:**
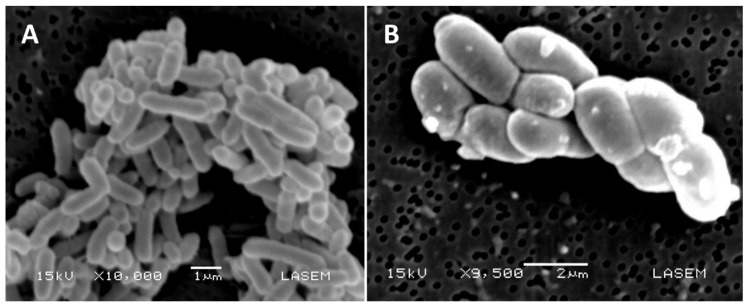
Scanning electron microphotographs of two different strains of *Streptomyces* spp. isolated from soils contaminated with boron compounds in Salta (Argentina): *Streptomyces* sp. 133 (**A**) and *Streptomyces* sp. 043 (**B**). Cultures were grown in Casein Starch agar at 30 °C for 48 h to collect the samples of aerial mycelia for imaging.

Specific cell division triggers sporulation, a completely different process of cell division between vegetative and aerial hyphae. While in the first case, there is an irregular formation of the septa in vegetative hyphae with transverse walls that divide them into multigenomic compartments [29], in the second one, many septa form simultaneously and symmetrically. This process is followed by the development of spore compartments and cell fission, resulting in spore chains that have a single copy of the chromosome [29] (Figure 2).

In most bacteria, two daughter cells result from a mother cell by binary fission. However, in *Streptomyces*, cell division is not required for growth since, after a coordinated cell division event, the long aerial hyphae differentiate into spore chains consisting of up to 100 septa, leading to the production of haploid spore chains [29]. This process depends on the generation of sufficient FtsZ (filamenting temperature-sensitive mutant Z) protein in the development of the “early” regulatory genes *whiA*, *whiB*, *whiG*, *whiH*, *whiI*, *whiJ* [45] to support sporulation, and the transcription of *ftsZ*. FtsZ accumulates and locates forming ladders in partitions that subsequently delimit the spore compartments. This is followed by condensation and chromosomal segregation that ends with the closure of the septum and maturation of the spores [30].

Although in sporulation, the specific cell division is similar to bacterial binary fission, there is a drastic difference in how the location of the septum is controlled in *Streptomyces*, since it involves proteins specific to actinomycetes [29], which implies a great difference between aerial and vegetative cell division.

Given the hyphal rather than planktonic growth of *Streptomycetes* [46], their cytoskeleton and life cycle are much more complicated than that of most other bacteria. Moreover, recent research depicts a new behavior of *Streptomyces* when facing specific biotic (fungi) and abiotic (amino acids, decrease in carbon source) stress, inducing communication through volatile organic compounds and a novel mechanism within their life cycle acknowledged as hyphal exploratory growth [47].

### 1.3. Streptomyces Applications 

**Figure 3:**
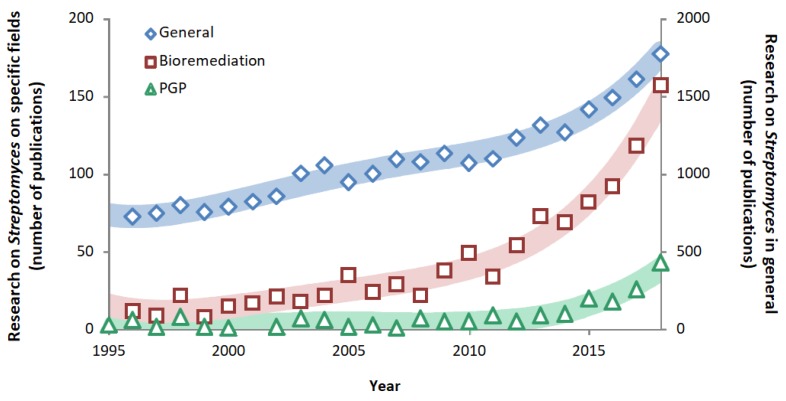
Number of research publications on *Streptomyces* spp. in general (secondary vertical axis), i.e., in all scientific fields (blue diamonds), and specifically (main vertical axis) in bioremediation (red squares) and plant growth promotion (PGP) (green triangles). Information based on the search algorithm from the ScienceDirect database [49]. The number of documents includes review and research articles, book chapters, case reports, mini-reviews, and short communications. Trendlines adjusted to R^2^ > 0.9 with fourth-order polynomial equations.

Since the discovery of streptomycin in 1944 [48], the study of *Streptomyces* became more relevant in the field of medical sciences [19]. However, in the last 15 years (2004–2019), the interest in the study on *Streptomyces* in the field of bioremediation increased. Moreover, the efforts of research in *Streptomyces* involved in PGP showed remarkable growth in the last five years (2014–2019) (Figure 3) [49]. Although these trends in research show the need of restoring a damaged environment that was once healthy, it is encouraging to note that efforts are being made to use the natural resources efficiently and sustainably to face the current and future needs of the growing world population.

*Streptomyces* have a set of suitable properties within their biochemical toolset regarding bioremediation and plant growth (Table 1). Most *Streptomyces* are non-pathogenic. Within their colonization strategies, they are also known now to be able to traverse solid surfaces [50], and they are prolific producers of versatile secondary metabolites. Compared to other microorganisms in the soil, members of the genus *Streptomyces* seem to be superior candidates for bioremediation and bio-reclamation due to their high tolerance and adaptability to different environmental stress conditions. As example, *Streptomyces coelicolor* genetic studies [51] meant an important turning point that revealed almost 8000 genes with more than 20 groups capable of directing the biosynthesis of “secondary” or “specialized” metabolites, (including antibiotics and pigments) as well as other compounds of unknown functions. This not only doubles those discovered for *E. coli*, but considerably more than *Saccharomyces cerevisiae* (to our knowledge, the only eukaryotic microorganism sequenced to date) and shows that the genome contains information to synthesize many more unknown molecules according to the stimuli (chemical, physical, or biological) of their environment [52].

## 2. *Streptomyces* in Bioremediation 

Bioremediation involves the use of organisms and or their metabolic activities to eliminate, reduce, or transform contaminants in species less deleterious to the environment [61]. Briefly, contaminant compounds are sequestered or transformed by living organisms through enzymatic pathways that take place as a part of their metabolic processes, generally as the result of multiple organisms’ actions [62]. In this sense, the use of microorganisms has multiple advantages as they are ubiquitous on the biosphere, their metabolic ability, and nutritional versatility is impressive, and they can multiply in a wide range of environmental conditions [63]. Additionally, the use of relatively low-cost, and low-technology techniques, generally has good public acceptance and can often be carried out on-site [62].

In the last 15 years (2004–2019), bioremediation mediated by *Streptomyces* became very important, especially in the case of hydrocarbons [56], organochlorine compounds used in agriculture [64,65,66,67,68,69,70], heavy metals [71,72,73,74,75,76,77], and in the reclamation of environments with naturally high concentrations of salt or toxic compounds such as boron [6,78,79]. 

### 2.1. Microbial Mechanisms Used for Bioremediation

Microorganisms developed numerous mechanisms to survive in toxic environments [80], to degrade and transform substances into less toxic compounds. Thus, *Streptomyces* can excrete toxic components through intracellular and extracellular transport systems, generate sequestering compounds that can bind and eliminate toxic agents from their interior and excrete extracellular chelating compounds to immobilize or solubilize toxic substances [66,81]. Microorganisms are also able to prevent the entry of toxic substances by adhering them to the cell membrane [82], and developing cytoplasmic protection mechanisms through inclusion bodies that retain a large number of toxic substances [81] to immobilize them. Besides, some microorganisms can immobilize toxic elements by forming biominerals with them inside or outside their cells [6,83,84].

**Figure 4:**
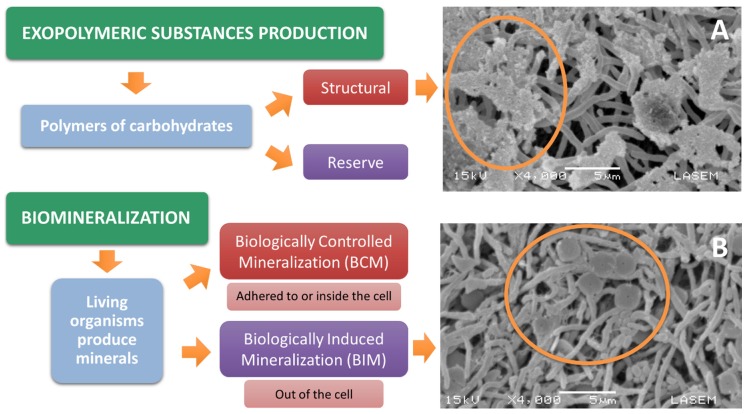
Bioremediation strategies (green rectangles) observed in two *Streptomyces* sp. strains isolated from soils contaminated with boron compounds in Salta (Argentina), grown at 30 °C and 250 rpm for 72 h in Minimal Medium with 40 mM of boric acid [6]. (**A**) Structural exopolymeric substances (EPS) formed by *Streptomyces* sp. 048, possibly to join or complex the boron present in the solution. (**B**) Biominerals formed by *Streptomyces* sp. 053 through a BIM mechanism as consequence of changes in the oversaturation due to the high concentration of boric acid in the medium. In the two-scanning electron microscope (SEM) images, circles mark both the EPS (A) and the biominerals (B) formed at 40 mM of boric acid as a defense mechanism to survive high concentrations of boric acid.

Biomineralization is the process by which living organisms produce minerals, being microorganisms the second most important group that produces a great variety of different minerals. Biomineralization can happen through two different mechanisms: biologically controlled mineralization (BCM) and biologically induced mineralization (BIM) [84]. There is evidence of biominerals produced by *Streptomyces* by active and passive mechanisms in BIM [6,22,83], where the formation occurs as a consequence of changes in the oversaturation of the system [85]. Active mechanisms comprise the capture or excretion of different metabolites [86]. Passive mechanisms involve the contribution of crystallization nuclei (cells or cellular components such as cell wall, membrane, organic lysis debris) that act as “seeds” for precipitation initiation [86] (Figure 4).

Another strategy of great importance in bioremediation is the bacterial production of exopolymeric substances (EPS), which are used by the microorganisms to remove toxins from the environment. Bacteria use compounds such as polysaccharides to aggregate the toxic components, immobilizing or flocculating them from a solution [87]. Exopolysaccharides are carbohydrate polymers of great structural diversity according to the different functions they have. Structural EPS such as cellulose are responsible for the interactions between cells and their cells with the surface [6] and their composition varies not only with the microorganism, but also with the environmental conditions that favor its growth [88]: availability of oxygen and nutrients, temperature, pH, and presence of stressors such as heavy metals in addition to high salt concentrations. Even when generated by the same species, EPS can have different compositions [6]. Polysaccharides are carbohydrate polymers whose different structural properties depend on their functionality: reserve (like glycogen) and structural ones (like cellulose). Their composition varies with the microorganism and with the environmental conditions under which they develop [89].

Within the group of actinomycetes, *Amycolatopsis* sp. AB0 stands out because it forms an EPS in the presence of copper [74], and in the extreme environment the “Salar del Hombre Muerto” actinomycetes with the ability to produce EPS in the presence of lithium were isolated [88]. Recently, Moraga et al. [6] isolated *Streptomyces* spp. strains from a natural environment with high boron concentration, which produce an EPS that allows them to tolerate and survive under those environmental conditions (Figure 4). 

As bioremediation refers to the application of biological systems to remove organic and inorganic contamination using microorganisms, decontamination through the immobilization of toxic compounds either by the formation of biominerals or EPS, can be an excellent way to recover polluted environments. 

### 2.2. The Case of Boron-Mining Environmental Impact

The northwest of Argentina is a reservoir of boron (B) minerals and lithium (Li) brines. The province of Salta in particular, ranks as the first Latin American borates producer, the first world producer of hydroboracite, and the third world producer of borates [90]. In fact, between 2001 and 2016 the production of borates (considering all the compounds) amounted 535,660 tons, reaching in 2016 its highest annual production value with 148,390 tons, i.e., 30% of the total production in 15 years [90].

However, the mining activity related to these minerals not only has a significant impact on the economy but also on the environment. There are places contaminated with boron compounds in Salta; Tincalayu, an exploitation mine of boron minerals in the west of the province; and Animaná, where pollution comes from natural leaching of bedrock in the Calchaquíes Valley. Another contaminated site, Baradero, where boron minerals were processed in an industrial plant, is in the middle of Salta City [79].

Numerous microorganisms were isolated from these polluted soils in Salta province. Seven of them were sequenced and genetically identified as *Streptomyces* spp. [78], showing > 99% homology with some species already identified and also classified as halotolerant (according to the criteria of Zahran [91], since they were able to tolerate up to 5 % w/v NaCl): *S. achromogenes* strain 048 (GenBank: HQ538723.1), *Streptomyces* sp. 133 (GenBank: HQ538729.1) closely related to *S. griseosporeus* (98.5% homology), *S. albogriseolus* strain 053 (GenBank: HQ538724.1), *S. iakyrus* strain 002 (GenBank: HQ538731.1), *S. ambofaciens* strain 002 (GenBank: HQ538725.1), *S. polychromogenes* strain 002 (GenBank: HQ538727.1), *S. lincolnensis* strain 128 (GenBank: HQ538726.1).

To analyze the strains’ resistance and tolerance to boron, they were cultured in liquid media with 20 and 40 mM of boric acid and subsequently evaluated in contaminated soils for their potential use as soil remediation agents. Through these studies, two of these strains (*Streptomyces* sp. 053 and *Streptomyces* sp. 182) showed that they could grow in high concentrations of boron through the formation of a biologically induced biomineral, while two others (*Streptomyces* sp. 002 and *Streptomyces* sp. 048) revealed the formation of EPS in liquid medium (Figure 4) [6]. Energy dispersive X-ray spectroscopy (EDS) microanalysis of the biominerals produced by both strains revealed that, regardless the microorganism, all the structures had high contents of boron and oxygen in their composition [6].

## 3. *Streptomyces* in Plant Growth Promotion

**Figure 5:**
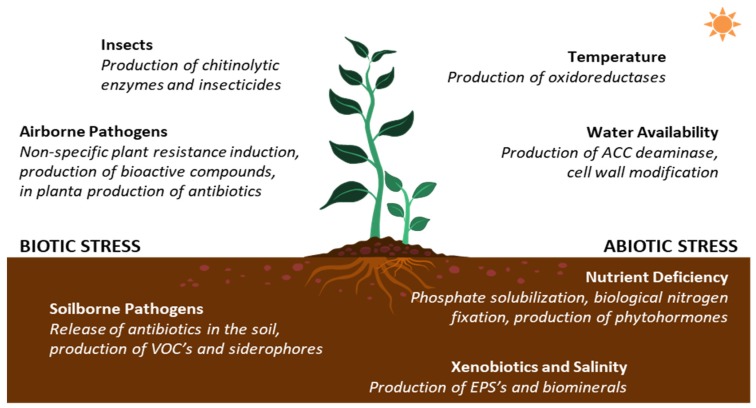
Schematics of counteracting mechanisms (italics) provided to plants by plant growth-promoting microorganisms against biotic (left side) and abiotic (right side) stressors (in boldface). VOC’s: volatile organic compounds, ACC: 1-aminocyclopropane-1-carboxylate, EPS’s: exopolymeric substances.

In general, PGP mechanisms are mediated by microorganisms and are described to have a direct or indirect effect on the plant’s growth [92]. Overall, these mechanisms enhance the plants’ performance by participating in their interaction with the biotic and abiotic factors of the growth environment. Briefly, different bacterial PGP mechanisms provide plants with defenses against different biotic and abiotic stress factors (Figure 5).

Through microbial PGP, plants benefit from the interaction with their surroundings. If soil and climate are appropriate for growth, environmental conditions can be enhanced (e.g., higher nutrient availability) [14,15]. However, if the environmental conditions are hostile (salinity, drought, etc.), PGP mechanisms can aid plants to endure stress and improve their survival under limiting conditions [13,93,94,95,96,97,98]. 

Regarding interactions with other living organisms, PGP microorganisms can elicit non-specific plant defense mechanisms against pathogens and can act as biocontrol agents, preventing the attack of pathogens and predating insects through antibiotics and insecticides before they come in contact with the plants [99,100,101,102,103].

### 3.1. PGP Streptomyces against Biotic Stressors

To date, less than 15 species of *Streptomyces* were reported as plant pathogens: *S. acidiscabies*, *S. aureofaciens*, *S. bottropensis*, *S. cheloniumii*, *S. europaeiscabiei*, *S. ipomoeae*, *S. luridiscabiei*, *S. niveiscabiei*, *S. puniciscabiei*, *S. reticuliscabiei*, *S. scabies*, *S. stelliscabiei*, and *S. turgidiscabies* [104,105,106,107]. Most of the mentioned pathogenic *Streptomycetes* cause scab disease on tubers, producing important economic losses on potato production [104]. In a recent review about virulence mechanisms of plant-pathogenic *Streptomyces*, such mechanisms were grouped under three main categories: phytotoxins (phytotoxic specialized metabolites), phytohormones (small molecules that alter plant hormone signaling), and effectors (proteins secreted into the plant promoting pathogenesis) [106]. Regarding horizontal gene transfer, current knowledge indicates that mobilization of the thaxtomin biosynthetic gene cluster can lead to the emergence of new pathogenic species, because thaxtomins are essential pathogenicity determinants in *Streptomyces* spp. [106]. However, new plant pathogens are infrequent in agricultural systems [104]. Moreover, some *Streptomyces* can interact in synergy with other PGP microorganisms in food, cash, and horticulture crops. In the case of peas (*Pisum sativum*), the strain *Streptomyces lydicus* WYEC108 influences *Rhizobium* spp. root nodulation. By colonization of the roots, the actinobacteria increase the size of the nodules, improving iron and nutrient assimilation, which benefits the nitrogen-fixing bacteria that live inside them [11]. However, the major traits of Actinobacteria are the production of a wide variety of bioactive compounds [108] and many well-known mechanisms by which they inhibit plant pathogens in the soil. Some of these mechanisms include antibiosis, nutrient competition, quorum quenching, and production of degradative enzymes and nitrous oxide [109,110].

The adaptability of *Streptomyces* to different environments in the rhizosphere, based on secondary metabolites production, makes them strong competitors. Some are known for their siderophores’ production, which can chelate iron, depriving other organisms of this vital micronutrient. Such is the case of *S. griseorubiginouse* siderophores against *Fusarium* [111,112]. Additionally, species like *S. antibioticus, S. aureofaciens* [113], *S. lividens* [114], *S. plicatus* [115], *S. halsteii* AJ-7 [116], and *S. lydicus* WYEC108 [117], secrete enzymes that degrade the mycelial cell walls of fungal parasites [108,118,119]. Moreover, some species produce antibiotics that allow them to inhibit plant pathogens [108,120,121]; for example, *S. violaceusniger* YCED9 produces three antifungal compounds (nigrecine, geltanamycine, and guanidylfingine) that fight against plant pathogens [119].

Most *Streptomyces* thrive in free life in the soil, providing the root surface of plants, when colonized, an outer barrier against soilborne pathogens. However, endophytic strains also protect some plants against threats by releasing antibiotics inside the plant’s tissue [100,101]. In some cases, bacteria can act in a vaccine-like fashion, eliciting non-specific plant defense mechanisms. This effect provides the host with resistance against pathogens via different mechanisms along the plant infection cycle [122,123,124]. Additionally, the use of some strains in the production of insecticides renders and environmentally friendly approach to control pests and disease without the application of synthetic compounds [99,102].

### 3.2. PGP Streptomyces against Abiotic Stressors

Plants are sessile organisms, once anchored to the soil in a specific location; they cannot outrun detrimental environmental conditions. Hence, in the face of water and nutrients scarcity or the presence of toxic substances in the soil, they must manage in the best possible way to survive. Regarding underground strategies, plants explore the soil with their roots, searching for what they need and avoiding what can harm them [125].

Many mechanisms participate in alleviating plant stress caused by nutrient deficiency. When bacteria facilitate plant uptake of soil nutrients or supply the plant with a bacterial-synthesized compound, direct plant growth promotion occurs [92]. Nutrients such as nitrogen and phosphorus limit primary production. Hence, bacteria that provide plants with these elements act as biofertilizers. Nitrogen-fixing microorganisms reduce atmospheric nitrogen into ammonia using a nitrogenase enzyme complex. To date, the known nitrogenase systems are molybdenum nitrogenase (Nif), vanadium nitrogenase (Vnf), and iron only nitrogenase (Anf). The different systems are similar in their nitrogen fixing mechanism and sensitivity to oxygen. However, they differ in their metal content and encoding genes; *nifH* being the most studied and conserved nitrogenase enzyme complex-encoding gene [126]. Only *Streptomyces thermoautotrophicus* was once believed to fix atmospheric nitrogen; however, MacKellar et al. [127] demonstrated its lacking nitrogen-fixing ability. However, recently, serendipity led to the finding of five nitrogen-fixing *Streptomyces*. The strains yielded *nifH* products that aligned with those of *Scytonema*, *Nostoc*, and *Bradyrhizobium* [126]. However, the nitrogen-fixing activity remains to be studied further in *Streptomyces*.

Regarding phosphorus, in order to make it available for plant use, microorganisms transform it from the immobilized organic and inorganic forms into soluble phosphates. Via phytases, microorganisms such as *Streptomyces* sp. and *S. luteogriseus* R10, catalyze the hydrolysis of phytates into myo-inositol and phosphate, showing optimal activity around 45–55 °C [128,129]. Other strategies used by *Streptomyces* sp. strains to solubilize phosphate from inorganic phosphorus sources involve the production of organic acids such as gluconic [130] and malic [131] acids. *Streptomyces* can also improve the plants nutrients uptake indirectly, by producing phytohormones that stimulate the growth and development of roots and root hairs. Such is the case of auxins like indole-acetic-acid (IAA), which by promoting the expansion of the root network, allow plants to explore more soil underground, and also to have a higher uptake surface, improving water and nutrients recollection efficiency. Information about hormone producing *Streptomyces* is scarce. However, several *Streptomyces* spp. isolated from marine environments, produced gibberellic acid, IAA, abscisic acid, kinetin, and benzyladenine [132]; all of them hormones with promising activities to be tested in plants in the future.

Unlike most animals, plants cannot regulate their temperature. Instead, over time, they developed physiological mechanisms that allowed them to adjust, and in some cases, tolerate temperatures (higher and lower) beyond their optimal growth conditions [133]. High and low temperatures have different detrimental effects on crops, evidenced as yield and productivity losses, which have a direct financial negative impact on farmers. Moreover, temperature variations over the last century already showed distribution shifts in animal and plant populations, following appropriate conditions to their physiological needs [134]. Thus, if global weather keeps changing, defenses against stress sources related to water availability and temperature variation will need to be addressed in plants. Many attempts are made seeking to alleviate temperature stress on crops, adequate nutrient managing being the most promising strategy [133]. Hence, the proper control of nutrients availability mediated by bacteria could be a required field to explore in the future. Additionally, the synthesis of compounds like ACCD (1-aminocyclopropane-1-carboxylate deaminase), which interferes and reduces the synthesis of ethylene [58,135] or antioxidant enzymes [136,137] that help plants to cope with the damage of reactive oxygen species (ROS) [57], is a vast field to explore further in plant abiotic stress alleviation mediated by *Streptomyces*.

Studies related to plant-microbe interaction in the frame of climate change are scarce. Current works involving *Streptomyces* focus on drought tolerance [60,95,96,138], and incipient efforts are made to analyze possible defense mechanisms in flooded soils [135]. Root microbiomes ecology in flowering plants shows that drought events cause a shift in the microbial community composition towards *Actinobacteria* and in particular, the abundance of *Streptomyces* associated with the host’s tolerance to drought [139]. Moreover, drought remodeled sorghum root microbiome through a plant-bacteria cross-talk. Plants under water-stress modify their normal root exudates, favoring the abundance and activity of *Streptomyces* related to drought tolerance [60]. Still, there is a critical gap yet to be filled in the field regarding the alleviation of plant stress caused by climatic factors (variation of temperature, light, water availability, etc.).

As bioremediation agents, *Streptomyces* (in particular halophiles), participate in alleviating the toxic effect of xenobiotics on plants, using the strategies previously mentioned. *Streptomyces* are widely used in the remediation of soils contaminated with different pollutants. Moreover, they are studied as PGP in salt-affected soils, where they provide plants with salt resistance to face this major problem for agriculture [58,82].

## 4. Bio-Reclamation of Saline Soils

All soils contain some amount of soluble salts that act as a source of nutrients for the plants. However, when the concentrations exceed a particular value, salts affect most crops adversely to different degrees, depending on the type and amount of salts present, the plant species, and their growth stage, and other environmental factors [140]. These adverse effects decrease the fertility and productivity of the soils, rendering them unsuitable for cropping.

### 4.1. Soil Salinity, Causes and Effects

Salt-affected soils contain soluble salts or their ions (at least in one horizon) in concentrations above the threshold of toxicity, i.e., the maximum permissible concentration of salts that do not suppress plant growth [141]. 

**Figure 6:**
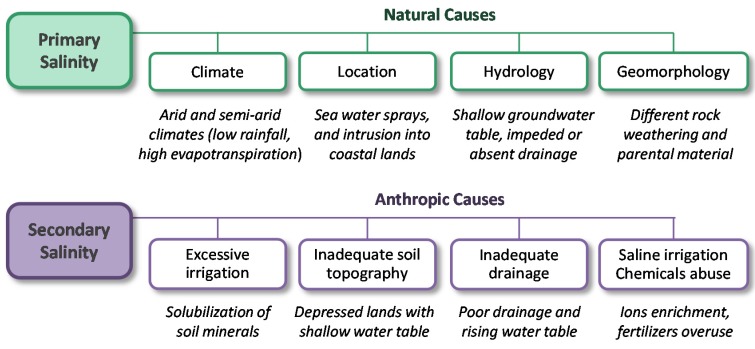
Types of soil salinity and its leading causes. Primary salinity, caused by a combination of natural factors, and secondary salinity produced by anthropogenic causes.

There are two main types of soil salinity, primary and secondary. Primary salinity occurs by a natural salt accumulation in the soil, driven by environmental conditions (topography, mineral composition of the earth’s surface, rain, groundwater depth, etc.). Conversely, secondary salinity has anthropogenic causes as inadequate management in the face of deficient environmental conditions for agriculture, excessive use of fertilizers, or poor management of irrigation water: use of saline water or merely the excessive input of water in the absence of proper drainage [141] (Figure 6). 

Salinity causes two main problems in the soil. First, the increase of salt content in the soil solution raises the osmotic pressure, favoring intense competition with the roots for the available water. Secondly, the saturation of the soil exchange complex with sodium collapses the soil’s structure, generating impermeable layers, and alkalizing the soil solution [142]. In this sense, salts affect three significant aspects of the soil: structural properties, ecological and environmental soil quality, and the soil’s agricultural production [143].

Overall, salt-affected soils present low productivity, and salinity effects depend on many factors such as climate, soil conditions, agronomic practices, irrigation management, crop type and variety, stage of growth, and salt composition [143]. In general terms, saline stress in plants is characterized by two main phases. First, osmotic stress caused by water deficit produces a decrease in growth, the first typical effect of salinity [144]. Soluble ions such as Cl^−^, SO_4_^2−^, HCO_3_^−^, Na^+^, Ca^2+^, Mg^2+^, and sometimes NO_3_^−^ and K^+^ can harm plants by reducing the osmotic potential. As water content decreases through evaporation and transpiration, water potential decreases and becomes more negative [145], causing an osmotic pressure effect [143]. In the second phase, ion-effect dominates, initiating and accelerating plant senescence and causing toxicity and mineral nutrition problems in some cases. 

The main salinity effects in plants can be seen in growth, photosynthesis, signaling, hydraulic/stomatal conductance, and oxidative stress response, among others [144]. Ionic stress is also related to toxicity caused by high Na^+^ and Cl^−^ concentrations in the soil solution and can create mineral nutrition problems such as Ca^2+^ deficiencies, common when Na^+^/Ca^2+^ ratio is high in soil water [143]. These detrimental effects were observed in many agronomic and horticultural crops. Visual symptoms of salt injury in plant growth appear progressively. The first signs of salt stress are wilting, yellowed leaves, and stunted growth. In a second phase, the damage manifests as chlorosis of green parts, leaf tip burning, and blade necrosis, and the oldest leaves display scorching [146]. In broccoli, salinity causes growth reduction due to water deficit and to the accumulation of salts in the shoot at toxic levels [147]. In spinach, salinity showed a significant decrease in leaf number; also, Na^+^ and Cl^-^ increased while K^+^ decreased in leaf tissue. Salt accumulation also inhibited photosynthesis, one of the primary processes affected by salinity [148,149]. Salinity also reduces root biomass in broccoli and cauliflower [150]. Irrigation with saline water can also increase the occurrence of blossom end rot (a nutritional disorder related to Ca^2+^ deficiency) in tomatoes, peppers, and eggplants [146].

In the case of agricultural crops, corn (*Zea mays* L.) and soybean (*Glycine max* (L.) Merr.) (the most valuable cash crops worldwide), salinity effects depend on growth stage. Overall, during germination they are more tolerant than in later vegetative growth stages. In corn, reports showed decreases in height, dry matter, leaf area index, and leaf nitrogen content; in response to increasing soil salinity [151]. In the case of soybean, the growth stage tolerance is highly dependent on the variety. Effects of salinity include significant declines in height, leaf area, photosynthesis, nutrients uptake, root nodulation, and gas exchange, leading to a reduced efficiency of N_2_ fixation in legumes [152,153,154].

In summary, salinity can intensify adverse effects such as dispersion of soil aggregates, transport of organic and inorganic contaminants [155], erosion [155], mobilization of soluble salts into groundwater, release, and leaching of heavy metals into the soil solution [156,157] and can also promote shifts in the plant cover (e.g., from mesophytes to halophytes). All these adverse effects of salinity bring environmental and economic costs. There is a decline in farmer welfare as a result of higher costs of cultivation (loss of soil fertility which requires fertilizers input), lower revenues (decrease of yields, poor quality of crops), and higher maintenance costs (shorter life of infrastructure and machinery due to corrosion) [140].

### 4.2. Salt-Affected Soils Classification and Distribution

**Figure 7:**
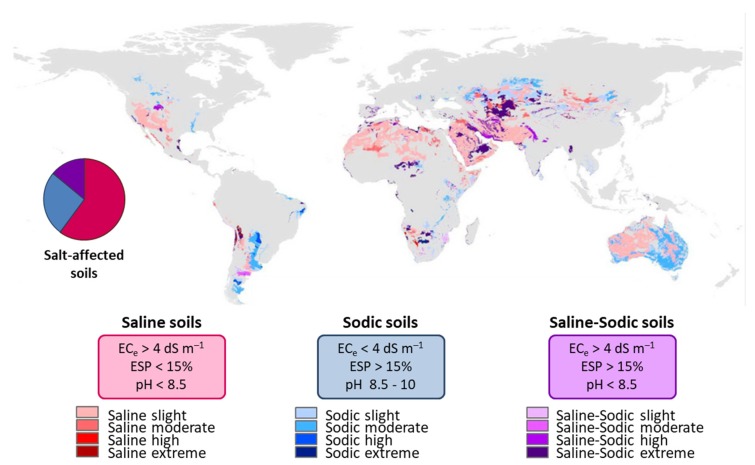
Classification and world distribution of salt-affected soils under different levels of saline stress. Saline soils (pink range) encompass 60% of the total salt-affected soils, sodic soils (blue range) include 26%, and saline-sodic soils (purple range) include the remaining 14%. Three main parameters are considered for the classification: electrical conductivity on the saturated soil extract (EC_e_), exchangeable sodium percentage (ESP), and pH (modified from Wicke et al., 2011 [158]).

Salt-affected soils classify as saline, sodic, and saline-sodic [143]. The three parameters considered for such classification are electrical conductivity of the saturated soil extract (EC_e_), exchangeable sodium percentage (ESP), and pH (Figure 7). Wicke et al. [158] mapped salt-affected lands under different grades of severity in the world (Figure 7). Most of the salt-affected lands correspond to saline soils (60%), followed by sodic soils (26%), and finally saline-sodic soils (14%). When considering the severity of the saline stress, 65% of salt-affected soils are slightly affected, followed by 20% moderately, 10% extremely, and 5% highly salt-affected soils (Figure 7).

The major problem in soils classified as saline is the high concentration of soluble salts as Cl^−^, SO_4_^2−^, and sometimes NO_3_^−^, and in lower proportion low-solubility salts, such as CaSO_4_ and CaCO_3_. In this case, exchangeable Na^+^ and soil clay dispersion are not a problem; therefore, saline soils maintain the structure of aggregates, and water permeability is good [143,159]. 

In sodic soils, Na^+^ is the major problem because high amounts of this cation along with low EC_e_ result in soil dispersion. Clay dispersion occurs when the electrolyte concentration decreases below its flocculation value [160]. These soils have weak structural stability and low hydraulic conductivity (HC) and infiltration rate (IR). These poor physical properties result in decreased crop productivity caused by poor aeration and reduced water supply. Historically, sodic soils were often called black alkali soils because sodium causes the dispersion of organic matter and dissolution of humic substances, which remain on the surface of the land resulting in a dark color [143].

Saline–sodic soils present both high soluble salts and exchangeable Na^+^. The soil maintains the aggregation with high electrolyte concentration. However, if soluble salts are leached out, usually Na^+^ becomes a greater problem since the soil pH rises above 8.5, and the soil aggregates can be dispersed [143,159].

Soil salinity is a problem that is spreading globally and is projected to increase in future climate change scenarios. Salinity problems occur under all climatic conditions and can result from both natural and human-induced actions. However, saline soils are more frequent in arid and semi-arid regions (Figure 7), where rainfall is insufficient to meet the water requirements of the crops and leach mineral salts into the root-zone [161].

Recent estimates of the global extent of soil salinization are not available. However, it is reasonable to assume that, since the data gathering in the 1970s and 1980s, salinization expanded and newly affected areas most probably exceed the areas restored through reclamation and rehabilitation [162]. According to the Harmonized World Soil Database (HWSD) [158,163], salt-affected land accounts for 1128 Mha (saline soils 60%, sodic soils 26%, and saline-sodic soils 14%; Figure 7). Salt-affected soils are found all over the world, although their extent and severity are variable (Figure 7). Regions with the largest salt-affected land areas are the Middle East (189 Mha), Australia (169 Mha), North Africa (144 Mha), and the former USSR (126 Mha) [158]. 

Considering only salt-affected land that is in use or has potential use for agriculture (excluding forest, wetlands, unsuitable land, high biodiversity areas, among others) Africa (295 Mha) and Asia (291 Mha) have the highest extensions (Table 2). In the world, 971 Mha are affected by salts (Table 2), an important area considering the increasing need of soils for agriculture as population growth is rising [164]. According to Food and Agriculture Organization (FAO) records [165], in 2011, the global population was 7.04 billion people, reaching 7.47 billion in 2016. Following this increasing trend, demographers are forecasting a population size of 8.01 billion for 2025 and 9.01 billion for 2050.

### 4.3. Reclamation vs. Bio-Reclamation of Salt-Affected Soils

Salinization is one of the most extensive soil degradation processes, which makes soils unproductive and endangers their potential use [166]. In this sense, in order to recover, reutilize and reduce runoff and erosion in saline and sodic soils, reclamation is a common practice [167]. Reclamation consists, in general terms, of the re-establishment of the original characteristics of a degraded land surface [168] and, in the case of saline soils, it involves the improvement of soil physicochemical and microbiological features by different strategies that reduce salts content. To remove the salts beyond the arable layer (0–20 cm depth), conventional mechanisms for the reclamation of salt-affected soils involve either washing them with water or adding a soluble calcium source for the replacement of exchangeable sodium by calcium [169].

Saline soils can be reclaimed by leaching them with good-quality water, i.e., with low electrolyte concentration. The water removes the salts from the root zone by solubilization. Successful reclamation aims to reduce salinity in the topsoil (45–60 cm depth) below the threshold values for the crop of interest [143,159]. Instead, for the reclamation of sodic soils, generally gypsum (CaSO_4_·2H_2_O) or CaCl_2_ is added to remove the exchangeable Na^+^, which is replaced by Ca^2+^ in the exchangeable soil complex. Then, the Na^+^ is leached out as a soluble salt (Na_2_SO_4_ or NaCl), and the Ca^2+^ improves the permeability of the soil [143]. Calcium is slightly larger than sodium in size but doubles the load density of sodium and stabilizes the charges on soil micelles, favoring soil aggregation, porous structure, aeration, and permeability. In comparison, when sodium covers the same surface area as calcium, it cannot balance the negative charges of the soil micelles, resulting in their repulsion and consequent soil structure collapse [142]. Sulfur and sulfuric acid can also be applied to correct a sodium problem in calcareous soils [143].

Saline-sodic soils reclamation involves a combination of the previously described techniques. A practical method is the saltwater-dilution, where the soil is first rapidly leached with high electrolyte water (high concentration of Ca^2+^ and Mg^2+^), and then after the removal of Na^+^, the soil is leached with water of lower electrolyte concentration to remove the excess of salts.

These mechanical and physicochemical methods are costly, considering the resource (water and calcium source) costs and availability [160]. However, the increasing interest in the use of microorganisms as bioremediation agents provides potentially sustainable tools for bio-reclamation of soils under different degradation events [170]. As saline soils constitute abiotic stress, physical and chemical methods are not cost-effective. Moreover, the availability of chemical amendments is a problem. In this sense, the use of halophilic bacteria includes the recovery of these soils by promoting vegetation growth (thus, indirectly increasing crop yields) [171] and by improving soil properties like water retention, aggregation, and regulation of the diffusion of carbon sources through exopolysaccharides and biofilm formation [172].

The biotic approach plant-microbe interaction to overcome salt stress has recently received considerable attention. It is one of the more efficient methods used for the bio-reclamation of salt-affected soils. It consists of a beneficial association between plants and microorganisms. In the case of bacteria, they improve the plant’s nutrient uptake and produce plant growth-promoting compounds, restoring soil quality at the same time [172]. 

Certain bacteria can improve the reclamation of salt-affected soils due to the production of EPS under stress conditions. EPS protects microorganisms from osmotic stress and fluctuations in water potential. It also enhances water holding and cementing in the soil, playing a vital role in the formation and stabilization of soil aggregates, increasing soil adhesion to roots [173], and in the regulation of nutrient and water flows across plant roots through biofilm formation [172,174,175]. Salt tolerance in plants depends mainly on the roots’ capability for a limited uptake of Na^+^ and Cl^−^, and continued uptake of essential elements, particularly K^+^ and NO_3_^−^ [176]. In this sense, EPS could also serve as useful tools for alleviating salt stress in salt-sensitive plants, probably due to a reduced passive flow of Na^+^ and lower availability of salts for plants [177]. This process relates to their potential to bind cations including Na^+^ [178]. With this cation-binding ability, a high population density of EPS-producing bacteria in the root zone would decrease the content of Na^+^ available for plant uptake. 

The use of halophilic bacteria in the recovery of saline soils with the biotic approach plant–microbe interaction rests on three main fundamentals [171]. First, microbial activity in saline soils may favor the growth of plants resistant to soil salinity. Second, bacteria could be used as bio-indicators in saline wells, reporting that well water is not saline but of good quality. Furthermore, third, through genetic manipulation (by incorporating genes from halophiles encoding crucial enzymes) wild type plants could adapt to growing in saline soil conditions [179].

The domain Bacteria includes many types of halophilic and halotolerant microorganisms, spread over a large number of phylogenetic groups [180]. The different branches of Proteobacteria contain halophilic representatives, often having close relatives that are non-halophilic. Similarly, halophiles are also found among Cyanobacteria [181], Flavobacterium-Cytophaga branch, Spirochetes, and Actinomycetes [171]. Regarding Actinomycetes, in a recent study we showed the presence of these phylum representatives in soils with high concentration of salts [89]. In this study, species belonging to the genus *Kocuria*, *Micrococcus,* and *Curtobacterium* showed tolerance to LiCl, EPS production, and capacity of pigment synthesis. The potential of these bacteria in the interaction with plants in saline soils is currently under study.

### 4.4. Streptomyces in Salt-Affected Soils

New strategies of bio-reclamation of salt-affected soils involve the use of salt-tolerant bacteria as bioremediation and plant growth-promoting agents. First, we have to considerer halophilic *Streptomyces* as a source of multiple metabolites: antibiotics production in high salt concentration and alkaliphilic conditions [182], biosurfactant production and heavy metal resistance activity [183], and enzymes production including cellulase, protease, chitinase, lipase, and β-1-3-glucanase, among others [15]. This genus is also used in biodegradation and bioremediation of hydrocarbons in saline environments [184]. Second, we should consider their use in salt-affected soils as plant growth promoters, to aid in settlement of crops that complement the process of bio-reclamation of salt-affected soils.

**Figure 8:**
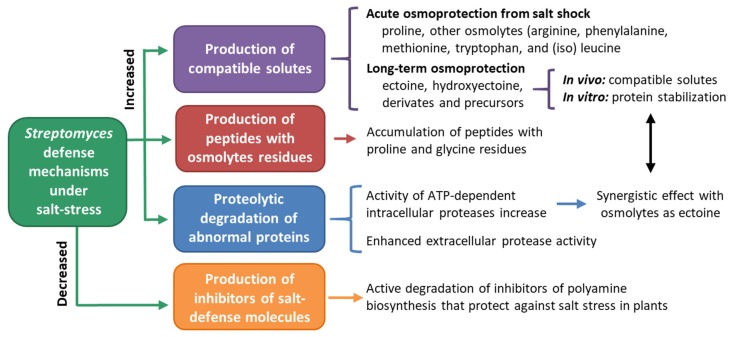
*Streptomyces* defense mechanisms against salts, which include the increased production of protective molecules and proteolytic degradation of anomalous proteins, as well as the decline in the production of inhibitors of salt-protective molecules.

In this sense, halophilic *Streptomyces*, and salt-tolerant bacteria in general, follow different mechanisms to tolerate and survive to saline stress. These mechanisms include the synthesis or uptake of various organic compatible solutes to balance the osmotic potential, including ectoine, alanine, glutamine, and proline [185,186,187] (Figure 8). 

Killhamt and Firestone [187] reported that *S. griseus* and *S. californicus* showed a marked change in the concentration and composition of the free amino acid pools with increasing salt stress: whereas concentrations of free glutamate and aspartate decreased, those of proline, glutamine, and alanine increased. This strategy was also seen in *S. parvulus* and *S. coelicolor*, with the production of ectoine and 5-hydroxyectoine biosynthesis, which serve as compatible solutes [185]. Furthermore, the metabolomic characterization of *S. coelicolor*, revealed the complexity of metabolite changes associated with salt stress response [188]. Under continuous salt exposure, ectoine, hydroxyectoine, and their precursors were found to accumulate strongly. However, under salt shock, the most important accumulating metabolite was proline, indicating that it is used for acute osmoprotection, while ectoine is used for long-term stress alleviation. In addition, other potential osmoprotectants were identified: arginine, phenylalanine, methionine, tryprophan, and (iso) leucine, which showed similar accumulation to proline and thus, could also play a role in acute osmoprotection. Moreover, during salt shock some metabolites as 5-methylthioadenosine showed a slight but consistent decrease. Since this acts as an inhibitor in the biosynthesis of polyamine (a known protectant against salt stress in plants) [189], 5-methylthioadenosine active degradation could be part of the salt response in *Streptomyces*. During salt stress, accumulation of peptides that contained proline and glycine residues were also detected [188]. 

Another stress response was seen in relation with abnormal and denatured proteins accumulated under stressful conditions. Since under various stress-related anomalous proteins are produced in higher quantities, they tend to be readily degraded by ATP-dependent proteolytic degradation [190]. Two families of ATP-dependent intracellular proteases have been well characterized: the Lon and Clp serine proteases [191]. In this sense, Sadeghi [190] reported increases of the *Streptomyces lon* mRNA levels under salt conditions. Moreover, the expression of *lon* in the presence of salt was increased even more in presence of ectoine, showing a synergistic effect of the protein protection systems. Furthermore, extracellular protease activity was also observed in salt amended media and enhanced after salt treatment in *Streptomyces* [190,192] (Figure 8).

Some *Streptomyces* strains produce different PGP compounds when they grow under salt stress. The mechanisms involve bio-fertilization and nutrients acquisition: siderophores production [13,15], tricalcium phosphate solubilization [93], and increase in the concentrations of N, P, Fe, and Mn in plants [13]. These beneficial compounds correlate with significant increases in plant biomass, number of lateral roots, and chlorophyll content, among other growth parameters [93]. 

Regarding biocontrol action and their possible use as biopesticides, soil isolated actinomycetes were tested for antimicrobial activities under different conditions of pH and salinity, showing the production of antimicrobial agents against a panel of bacteria, filamentous fungi, and yeasts, including some of clinical relevance [193]. In this sense, *Streptomyces* isolated from saline soils, produce secondary metabolites with antifungal and antibacterial activities [194]. They are efficient against root rot diseases [195] and also have potential for biocontrol of *Fusarium* wilt in chickpea, caused by *Fusarium oxysporum* f. sp. *ciceri* [196]. *Streptomyces* is the genus with the highest productive capacity of compounds with antibacterial or antifungal activity. Additionally, some produce hydrocyanic acid [15], and phytostimulation should be considered as well. The production of phytohormones alters the physiology of plants, which are then able to cope with abiotic stresses such as drought and salinity [197], whereas production of volatile substances by rhizobacteria regulates genes involved in sodium-ion homeostasis and protects plants from salinity stress [198]. The genus *Streptomyces* produces IAA and other auxins in the presence of salt [13,93]. Apart from ACC deaminase activity, rhizobacteria exhibit other mechanisms to alleviate abiotic stress in plants, such as the production of cytokinin and auxin, antioxidant enzymes (catalase), and volatile substances [197].

In all cases, these PGP traits improved germination rate, roots number, and uniformity, shoot length and dry weight and increased the concentration of nutrients in different crops showing the potential to use *Streptomyces* as biofertilizer in saline soils.

## 5. Concluding Remarks

Bacteria have been used since ancient times in a variety of ways in food industry, agriculture, pharmaceutical and chemical industry and, more recently, in bioremediation, due to the production of valuable compounds, the ubiquitous distribution, and high growth rates and metabolic diversity, among others [199]. In this sense, microbial diversity and biotechnology have been always strongly related.

The inhibition of pathogens responsible for plant diseases is sought in agriculture using antibiotics. However, the diversity of secondary metabolites produced by *Streptomyces* should also be considered as very useful to suppress fungi, bacteria, oomycetes, and nematodes. The prospects for improving agriculture by using PGP *Streptomyces* beyond biocontrol seem to be excellent, as interaction between microbial species and their plant symbionts appears to be specific [200]. However, because of the later, it should be kept in mind that a microorganism screened for growth promotion can have either a neutral, positive, or even negative effect on different crops [201,202]. Moreover, summed to the *Streptomyces* classical growth cycle (hyphae growth, aerial hyphae development, spore formation), the mechanism of “exploratory growth” has great potential in agriculture. This mechanism provides *Streptomyces* with the possibility of colonizing new environments, without compromising resources in terms of sporulation [50]. 

Salt toxicity in the soil is a major restriction for agriculture and also a limiting factor for bioremediation by non-halophile or halotolerant microorganisms due to the negative effect of salinity upon several soil enzymatic activities [203]. Saline soils occur naturally and by manmade contamination, in addition, they are often polluted by organic contaminants [204] and heavy metals [205]. Removing these components presents a challenge as they are resistant to degradation and even more in soils with high pH and large salt concentrations [206].

To face these constraints in land availability for cropping, it is required to reclaim soil surface. Frequently, soil reclamation methods are costly and, in the process, they demand the use of resources such as good-quality water in exchange of a waste product (saline water). Hence, there is a need to recover cultivated land surface in a sustainable way. Bio-reclamation aims in that direction and microorganisms such as *Streptomyces* seem to hold many traits that turn them into good suitors for the job. The combination of plant growth promotion traits with the capacity to tolerate the environmental conditions of salt-affected soils (augmented with many other metabolic activities, such as production of EPS, biominerals, antibiotics, priming compounds, etc.) grants *Streptomyces* the potential for bio-reclamation of salt-affected soils in the near future. Furthermore, if the products thus formed by the microorganisms in these conditions have novel and important properties, their study may impact the development of nanoparticles to expand the use of biomaterials with structural, technological, and environmental implications [207,208,209,210].

## Figures and Tables

**Table 1:** Recent research on *Streptomyces* sp. (from 2017 to date) suitable for bioremediation and agricultural purposes.

Potential	Species	Characteristics/Purposes
**Bioremediation**		
	*Streptomyces albogriseolus* 053 HQ538724.1 and *S. lincolnensis* 128 HQ538726.1	Formation of boron minerals by the cells [6]
	*Streptomyces* sp. DPUA1566	Production of a new biosurfactant lipoprotein for use in agro-industrial waste [53]
	*Streptomyces* sp. Hlh1	Degradation of petroleum compounds in contaminated soils [54]
	*Streptomyces* sp. strain M7	Possible lindane degradation [55]
	*Streptomyces antioxidans* MUSC164T	Remediation of soils chronically contaminated with hydrocarbons [56]
**Plant Growth Promotion**		
	*Streptomyces* T5	Increase of superoxide dismutase, catalase and phenol peroxidase activities in nodules of cowpea plants exposed to salt stress [57]
	*Streptomyces* sp. GMKU 336	Increase of salt-stress resistance of *Oryza sativa* L. cv. KDML105 [58]
	*Streptomyces* spp.	Increase of salt tolerance of Stevia [59]
	*Streptomyces coelicolor* (Sc1) and *Streptomyces ambofaciens* (Sc2)	Colonization of roots during drought to improve plant growth [60]

**Table 2:** Distribution of world population and land areas affected by salts and assigned to agricultural uses.

Region	Population (Millions)	Land Area with Irrigation ^a^ (thousand ha)	Arable Land ^b^ (thousand ha)	Permanent Crops ^c^ (thousand ha)	Salt-Affected Land ^d^ (thousand ha)
World	7043	324,548	1,395,490	162,100	971
Africa	1077	15,265	230,862	33,571	295
North America	346	27,730	194,640	7526	63
Central America	163	7306	28,195	5178	4
South America	400	15,880	133,326	14,199	57
Asia	4240	228,667	480,140	83,495	291
Europe	738	25,414	274,151	15,211	2
Oceania	37	3261	48,702	1603	144
Former USSR					117

^a^ Land area equipped with irrigation infrastructure and equipment to provide water to crops. ^b^ Total of areas under temporary crops, temporary meadows and pastures, and land with temporary fallow ^c^ Land cultivated with long-term crops. ^d^ Salt-affected land excluding forest, wetlands, unsuitable and high biodiversity areas.
